# Trajectories and risk factors for long-term breast symptoms following breast-conserving surgery and radiotherapy: a single centre analysis

**DOI:** 10.1007/s00520-025-10276-4

**Published:** 2026-01-09

**Authors:** Catherine Jones, Nur Nurmahomed, Monika Kaushik, Jaroslaw Krupa, Kelly V. Lambert, Simon M. Pilgrim, Kiran Kancherla, Kufre Sampson, Walid Sasi, Petra Seibold, R. Paul Symonds, Kalliope Valassiadou, Adam J. Webb, Catharine West, Christopher J. Talbot, Tim Rattay

**Affiliations:** 1 Leicester Cancer Research Centre, Department of Genetics and Genome Biology, University of Leicester, Leicester Royal Infirmary, Leicester, LE1 5WW UK; 2 Department of Breast Surgery, University Hospitals of Leicester NHS Trust, Leicester, UK; 3 Department of Oncology, University Hospitals of Leicester NHS Trust, Leicester, UK; 4 Division of Cancer Epidemiology, German Cancer Research Center (DKFZ), Heidelberg, Germany; 5 Department of Genetics and Genome Biology, University of Leicester, Leicester, UK; 6 Division of Cancer Sciences, University of Manchester, Manchester, UK

**Keywords:** Breast cancer survivorship, Quality of life, Radiotherapy side-effects, Breast pain, Multivariate analysis

## Abstract

**Purpose:**

Long-term breast symptoms (pain, sensitivity, swelling and skin problems) after breast cancer treatment can affect survivors' quality-of-life. The trajectory of breast symptoms over time and risk factors associated with their development are not well understood.

**Methods:**

This study built on the work of the international prospective REQUITE cohort study. Patients who underwent breast-conserving surgery and adjuvant radiotherapy (± chemotherapy) completed the EORTC-QLQ-BR23 questionnaire items relating to breast symptoms at four timepoints up to 24 months following radiotherapy. Patients at were re-contacted to complete additional psychometric questionnaires on different aspects of pain perception and the Hospital Anxiety & Depression Scale (HADS), with 237 respondents.

**Results:**

Average breast symptoms peaked on completion of radiotherapy but returned to levels equal to or below baseline by 24 months. Patients with more severe breast symptoms at baseline continued to have worse symptoms long-term. In multivariable mixed models, higher breast symptom scores were associated with smoking (p = 0.036), any analgesic use at baseline (p = 0.005), and post-operative haematoseroma (p = 0.034), while older age and use of intensity modulated radiotherapy (IMRT) were protective (p =  < 0.001 and p = 0.045 respectively). Psychometric questionnaire scores for life interference and pain severity perception were associated with persistently increased breast symptoms at 24 months on multivariable analysis, while anxiety (as determined by HADS) was associated on univariable analysis.

**Conclusions and implications for cancer survivors:**

This study identifies several risk factors for persistent breast symptoms including younger age, smoking, and post-operative haematoseroma. This particularly highlights the importance of smoking cessation and use of IMRT in women at higher risk of side effects.

## Introduction

Breast cancer remains the most common cancer in women worldwide and accounts for 15% of all new cancer cases in the UK [1]. Breast cancer survival has improved markedly, with current 10-year survival rates in the UK around 80%. In 2020, it was estimated that 600,000 women who had previously been diagnosed with breast cancer were still alive in the UK, with this number predicted to reach 1.2 million by 2030 [2]. As this community of cancer survivors continues to grow, it is important that research addresses issues relevant to their survivorship and quality of life [3].

Owing to earlier cancer detection and newer systemic treatment and surgical options, the majority of patients in the UK are now able to undergo breast-conserving surgery (BCS), usually followed by adjuvant radiotherapy [4]. However, breast radiotherapy is associated with several side-effects in the normal tissue. Acute side-effects such as breast erythema, oedema and ulceration occur within 90 days of treatment. Late side-effects such as breast sensitivity, chronic oedema, hyperpigmentation and itching occur more than 90 days after treatment and may persist in the long-term [5]. Exact rates of side effects in the acute and late phase are unknown, but breast pain is the most frequently reported toxicity by patients. The reported prevalence of breast pain varies widely with an estimated 25% to 60% of patients affected [6] and a median prevalence of 37.5% reported in a systematic review [7]. Although its authors only included a limited number of high-quality studies in their meta-analysis, they showed that long-term pain after surgery was associated with younger age, radiotherapy, axillary lymph node dissection, and increased pain in the pre-operative and acute post-operative period [7].

There is conflicting evidence whether breast pain generally decreases with length of follow-up [8], or whether it may persist in some patients [9]. However, many previous studies lacked a formal definition for the different breast symptoms and were heterogenous in their design, with the use of multiple different assessment tools. For example, some examined pain in the acute post-operative period [10, 11], or in mastectomy patients only [12]. Nevertheless, factors commonly implicated in the development of breast pain in previous studies were patient age [7, 10, 13]; BMI [8]; breast size [14, 15]; axillary node dissection [10, 11, 16]; radiotherapy technique and fractionation schedule [17, 18]; and significant pre-operative breast symptoms [7, 10, 16].

Increasingly, cancer researchers have been collecting data on patient symptoms using standardised patient-reported outcome (PRO) questionnaires. It is well documented in radiotherapy trials that PROs correlate poorly with clinician-recorded outcomes (CROs) [19]. The aim of this study was to investigate patient-reported long-term breast pain and sensitivity following breast-conserving surgery and radiotherapy in a prospective patient cohort, its association with patient and treatment variables at baseline, and to explore the use of additional psychometric questionnaires to identify patients with increased long-term breast symptoms.

## Patients and methods

Breast cancer patients enrolled in the international multicentre prospective REQUITE cohort study at University Hospitals of Leicester (n = 237), who had completed PRO items relating to breast symptoms and pain at 2-year follow-up were re-contacted at least 36 months after enrolment to complete additional psychometric questionnaires. Patient inclusion criteria and methodology of the REQUITE study have been described in detail elsewhere [20]. All patients underwent breast-conserving surgery (with or without chemotherapy and endocrine therapy) and received external beam radiotherapy for breast cancer or in-situ disease according to local protocol and were recruited between 2014 and 2016. Mastectomy, partial breast irradiation or brachytherapy were excluded. In Leicester at the time, patients with invasive breast cancer were treated using a hypo-fractionated regimen (40 Gy in 15 fractions or 30 Gy in 6 fractions to the whole breast), with 11% of patients receiving a boost to the tumour bed, while patients with in-situ disease were treated with 50 Gy in 25 fractions. The majority (88%) of patients received forward-planned ‘field-in-field’ intensity-modulated radiotherapy (IMRT), and the remainder were treated by 3D-conformal technique. Patient demographic and treatment data were collected post-operatively at baseline prior to radiotherapy, and patients were assessed at end-of-treatment, and annually thereafter. All patients gave written informed consent. The REQUITE study and administration of additional psychometric questionnaires were approved by the relevant UK ethics committee (NRES 14/NW/0035) and registered at www.controlled-trials.com (ISRCTN 98496463).

### Outcome measures and endpoints

Breast symptoms were assessed using the European Organisation for Research and Treatment of Cancer’s Breast Cancer Module (EORTC-QLQ-BR23). This questionnaire comprises a set of 23 questions assessing multiple domains on a Likert scale, including body image, sexual function, side effects of treatment and breast and arm symptoms [21]. It has been validated in multiple countries and population groups [22, 23]. For the purposes of this study, four questions from the Breast Symptoms domain were used:

In the last week: (rate 1–4).

1. Have you had any pain in the area of your affected breast?

2. Was the area of your affected breast swollen?

3. Was the area of your affected breast oversensitive?

4. Have you had skin problems on or in the area of your affected breast (e.g., itchy, dry, flaky)?

The breast symptoms domain Raw Score (RS) is scored by calculating the mean scores of the component questions. Once the RS has been calculated, this is standardised using a linear transformation on a 0–100 scale, with higher scores reflecting more severe symptoms.

For the next stage of the study, patients who were still enrolled in the REQUITE study at University Hospitals of Leicester were re-contacted between 36 and 60 months after recruitment and mailed five psychometric questionnaires with a franked return envelope. Four questionnaires (Short Form McGill Pain Questionnaire, Pain Sensitivity Questionnaire, Pain Catastrophising Scale and West Haven-Yale Multidimensional Pain Inventory) were chosen in order to provide a detailed picture of the patients’ perception of pain and its current impact on their lives and their families, in keeping with the biopsychosocial model of chronic pain [24]. The fifth questionnaire was the Hospital Anxiety and Depression Scale, which was included because patients with chronic pain have also been shown to frequently suffer from underlying depression and/or anxiety [25, 26].

### Statistical analysis

All statistical analyses were carried out in Stata™ version 16.0 (StataCorp, College Station, TX, USA). P-values < 0.05 were considered significant. Descriptive statistics were calculated for each variable. Continuous variables were presented as median and range, while categorical variables were presented as frequency and percentage.

In order to investigate trajectories of breast symptom scores longitudinally over time, the baseline scores were split into quartiles. The median score for each quartile was calculated and plotted at each of the four time-points to allow visualisation of how patients in different quartiles of symptom severity progressed. EORTC-QLQ-BR23 Breast Symptom scores across all four timepoints were investigated in association with patient/treatment variables at baseline including patient age, body mass index (BMI), breast size, co-morbidities, co-medications, surgical and radiation therapy, and tumour stage, using both univariable and multivariable linear mixed models. As the additional psychometric questionnaire data were collected retrospectively, their association was investigated with breast symptom scores at the final available follow-up point (24 months) using linear regression.

## Results

Of the breast cancer patients originally enrolled in the REQUITE study in Leicester, 237 (68.1%) had completed the EORTC-QLQ-BR23 questionnaire at 24 months and were included in the analysis. The population demographics and treatment details of these patients are shown in Table 1. The median age of the participants was 60 (range 38–86), and the majority (92.4%) were White British. The most common co-morbidity was hypertension (32.9%), followed by depression (12.2%). Slightly less than half (44.7%) were current or ex-smokers. The majority were post-menopausal and 33.1% of all patients had been on HRT (hormone replacement therapy) prior to diagnosis. Most patients received hypofractionated radiotherapy (40 Gy in 15 fractions or 30 Gy in 6 fractions), 87.3% received simple field in field IMRT, and 23.6% of patients had chemotherapy. Haematoseroma was the most common surgical complication (13.3%).

**Table 1:** Demographics of 237 breast cancer study participants. Results are presented as the median and range for continuous data, and frequency and percentage for categorical variables

Variable	Results	Variable	Results
Age (years)	60 (range: 38–86)	**Tumour stage**	
BMI (Kg/m^2^)	26.4 (range: 13.1–48.1)	Tis	24 (10.3%)
Bra size (UK size)	36 C (range: 30A–44H +)	T1(a–c)	170 (73.0%)
Ethnicity White Indian Other	219 (92.4%) 13 (5.5%) 5 (2.1%)	T2	38 (16.3%)
		T unknown	1 (0.4%)
		**Tumour grade**	
		Well-differentiated	55 (23.2%)
Smoking status Never smoked Current/ex-smoker	131 (55.3%) 106 (44.7%)	Moderately differentiated	122 (51.5%)
Currently drinks alcohol	167 (70.5%)	Poorly differentiated	60 (25.3%)
Menopausal status		**Lymph node stage**	
Pre-menopausal	30 (12.8%)	N0	189 (80.8%)
Peri-menopausal	39 (16.6%)	N1	35 (15.0%)
Post-menopausal	166 (70.6%)	N2	10 (4.3%)
Co-morbidities		**ER status**	
Diabetes	19 (8.0%)	Positive	187 (78.9%)
Cardiovascular disease	17 (7.2%)	Negative	25 (10.6%)
Rheumatoid arthritis	3 (1.3%)	Unknown†	25 (10.6%)
Hypertension	78 (32.9%)	**Her2 Status**	
Depression	29 (12.2%)	Positive	22 (9.3%)
Co-medications		Negative	188 (79.3%)
HRT	55 (33.1%)	Neoadjuvant	27 (11.4%)
Anti-diabetic	13 (5.5%)	**Chemotherapy**	
ACE-inhibitor	30 (12.7%)	Adjuvant	43 (18.1%)
Other antihypertensive	66 (27.9%)	Anti-HER2 therapy	13 (5.5%)
Statins	41 (17.3%)	**Hormonal therapy**	16 (6.8%
Analgesic	32 (13.5%)	Tamoxifen	91 (38.4%)
Anti-depressant	30 (12.7%)	Aromatase inhibitor	76 (33.1%)
Breast surgery			
Segmentectomy/ quadrantectomy	17 (7.2%)	**Fractionation schedule**	
Wide Local Excision	220 (92.8%)	40 Gy in 15 fractions	205 (86.5%)
Axillary Surgery		50 Gy in 25 fractions	27 (11.4%)
No axillary surgery	21 (8.9%)	30 Gy in 5 fractions	5 (2.1%)
Sentinel node biopsy (SNB)	178 (75.1%)	**IMRT** No Yes	28 (11.8%) 209 (88.2%)
Planned axillary dissection	13 (5.5%)		
SNB followed by axillary dissection	25 (10.6%)		
**Post-operative complications** Haematoseroma Oedema Infection requiring any antibiotics Delayed healing*	30 (13.3%) 6 (2.8%) 24 (10.7%) 4 (1.8%)	**Radiotherapy** To axilla To SCF Boost Hotspots > 107%	19 (8.0%) 19 (8.0%) 26 (11.0%) 45 (19.0%)

All data was recorded at time of cancer diagnosis.

*BMI* body mass index, *HRT* hormone replacement therapy, *ACE-inhibitor* angiotensin-converting enzyme inhibitor, *SNB* sentinel node biopsy, *ER* oestrogen receptor, *Her2* human epidermal growth factor receptor 2. *Gy* gray, *IMRT* intensity modulated radiation therapy, *SCF* supraclavicular fossa.

*Delayed healing was defined as > 3 weeks following surgery.

†24 of the patients with unknown ER and Her2 status had carcinoma in situ and thus were not tested for Her2 and ER.

‡Hot spots were defined as “an area outside the planned treatment volume which receives a dose larger than 100% of the specified dose” [27]. This outcome has been binarized to those receiving over 107% of planned dose.

Sample size: 237 patients.

**Fig. 1:**
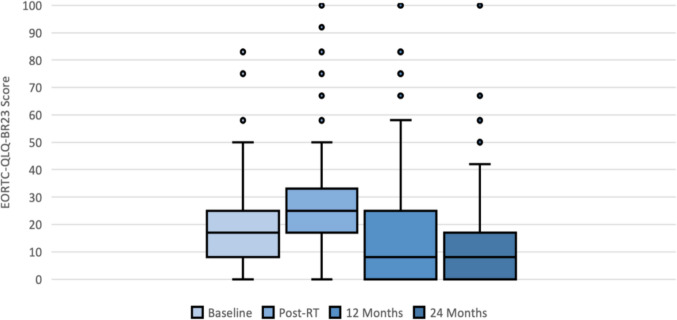
Box whisker plot illustrating distribution of EORTC-QLQ-BR23 breast symptom scores at baseline, post-radiotherapy, 12 months and 24 months (baseline: median: 17 (IQR 8–25), post-radiotherapy: median: 25 (IQR 17–33), range of data: 0–50, 12 months: median: 8 (IQR 0–25), range of data: 0–58; 24 months: median: 8 (IQR 0–17), range of data: 0–42). Sample size: 237 patients

Figure 1 demonstrates the distribution of breast symptom scores at four timepoints – baseline (post-surgery), immediately after completion of radiotherapy, 12 months, and 24 months. There were multiple outliers for each time point, showing that some patients had considerably higher symptom scores than the main cohort. The median scores decreased at 12 months to below baseline post-surgical levels, although the interquartile range increased to 25 (−0.99, 95% CI −3.24–1.25, p = 0.385). By 24 months, the interquartile range narrowed, and patients experienced an overall decrease in breast symptom scores (−2.08, 95% CI −2.90 to −1.27, p = 0.001).

**Fig. 2:**
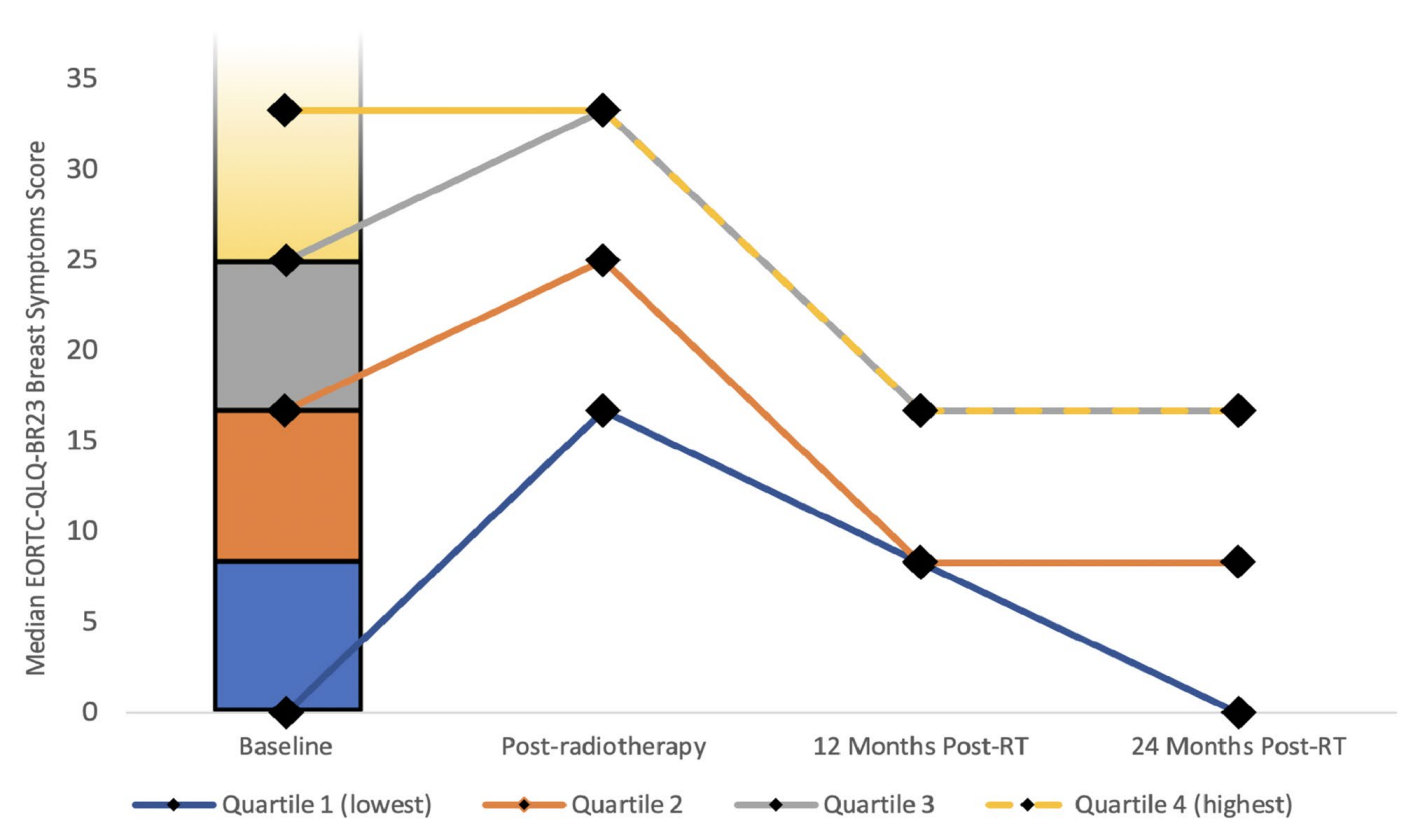
Graph illustrating how baseline EORTC-QLQ-BR23 breast symptoms scores change over time when split into quartiles. Baseline EORTC-QLQ-BR23 scores were split into four quartiles: quartile 1 (0–8.3), quartile 2 (8.31–16.7), quartile 3 (16.71–25), and quartile 4 (25–50). These quartiles were then plotted on a bar graph (quartile four is not shown in full). The median scores (diamonds) for each quartile at the four timepoints were calculated and plotted on a line graph. Quartile 1 median scores were 0, 16.7, 8.3, and 0. Quartile 2 median scores were 16.7, 25, 8.3, and 8.3. Quartile 3 median scores were 25, 33.3, 16.7, and 16.7. Quartile 4 median scores were 33.3, 33.3, 16.7, and 16. Sample size: 237 patients

Figure 2 shows the change in median breast symptom scores over time with patients split into four quartiles or trajectories according to baseline score post-surgery. Quartiles 1, 2 and 3 all peaked at the post-radiotherapy timepoint, while quartile 4 (those with the highest baseline scores) remained consistently high between the baseline and post-radiotherapy measurement, although it later reduced similarly to those in quartile 3. Patients in the lowest quartile showed the greatest increase from 0 at baseline to 16.7 post-radiotherapy, but this decreased over time and reverted to median zero baseline by 24 months. The scores for patients in the other three quartiles remained elevated above 0 at 24 months.

Table 2 shows the results of both univariable and multivariable analysis of breast symptom scores and patient/treatment variables over time. For brevity, only statistically significant variables have been shown here. A full table can be viewed in the Appendix. Younger age (beta −0.30, confidence interval −0.43 to −0.17, p < 0.001), increasing BMI (0.37, 0.15–0.59, p = 0.001), increasing breast size (0.71, 0.12–1.31, p = 0.019), pre-menopausal state (6.73, 2.42–11.05, p = 0.002), smoking (2.96, 0.19–5.73, p = 0.036), use of a statin (−3.91, −7.56 to −0.26, p = 0.036), the presence of hotspots > 107% of the prescribed dose (4.47, 0.90–8.03, p = 0.014), and receipt of radiotherapy boost (4.97, 0.42–9.53, p = 0.032) were significantly associated with long-term breast symptoms on univariable analysis. The protective effect of older age (beta = −0.28 per year, −0.41 to −0.15, p < 0.001) remained on multivariable analysis, while IMRT technique was also protective for long-term breast symptoms (−4.38, −8.66 to −0.10, p = 0.045). The effect of smoking (2.85, 0.18–5.51, p = 0.036) and use of analgesia at baseline (5.21, 1.56–8.86, p = 0.005) also remained significant. Additionally, the presence of post-operative haematoseroma was associated with increased breast symptoms on multivariable analysis (4.56, 0.35–8.77, p = 0.034).

**Table 2:** Association between EORTC-QLQ-BR23 breast symptoms score and patient/treatment variables over time. Values presented in this table are derived from multilevel mixed effects linear regression with EORTC-QLQ-BR23 scores – general trend over time. Multivariate regression was controlled for age, radiotherapy dose, axillary surgery type, BMI, smoking status, breast size, use of boost, and presence of hot spots. Ethnicity was analysed as a binary variable (white/non-white) due to small sample size of non-white participants

	Univariable analysis			Multivariable analysis		
Variable	Beta co-efficient	Confidence interval	*P*-value	Beta co-efficient	Confidence interval	*P*-value
Age (years)	** − 0.30**	** − 0.43** to − **0.17**	** < 0.001**	** − 0.28**	** − 0.41** to − **0.15**	** < 0.001**
BMI (Kg/m^2^)	**0.37**	**0.15–0.59**	**0.001**	0.20	− 0.08–0.48	0.155
Breast size*	**0.71**	**0.12–1.31**	**0.019**	0.42	− 0.33–1.17	0.275
Smoking	**2.96**	**0.19–5.73**	**0.036**	**2.85**	**0.18–5.51**	**0.036**
Pre-menopause	**6.73**	**2.42–11.05**	**0.002**	− 0.47	− 5.89–4.95	0.865
Statins	** − 3.91**	** − 7.56** to − **0.26**	**0.036**	− 1.61	− 5.35–2.13	0.400
Analgesic	3.59	− 0.20–7.38	0.063	**5.21**	**1.56–8.86**	**0.005**
Haematoseroma	3.78	− 0.48–8.05	0.082	**4.56**	**0.35–8.77**	**0.034**
IMRT	− 2.45	− 6.86–1.96	0.275	** − 4.38**	** − 8.66** to − **0.10**	**0.045**
Hot spots‡	**4.47**	**0.90–8.03**	**0.014**	3.13	− 0.42–6.68	0.084
Boost	**4.97**	**0.42–9.53**	**0.032**	2.84	− 1.70–7.38	0.220

*BMI* body mass index (Kg/m^2^).

*Breast size is measured in “sister sizes”—the band and cup size are added together to yield an estimate of cup volume.

*IMRT* intensity modulated radiation therapy, *RT* radiotherapy.

‡Hot spots are defined as “an area outside the planned treatment volume which receives a dose larger than 100% of the specified dose” [27]. This outcome has been binarized to those receiving over 107% of planned dose.

Sample size: 237 patients.

**Fig. 3:**
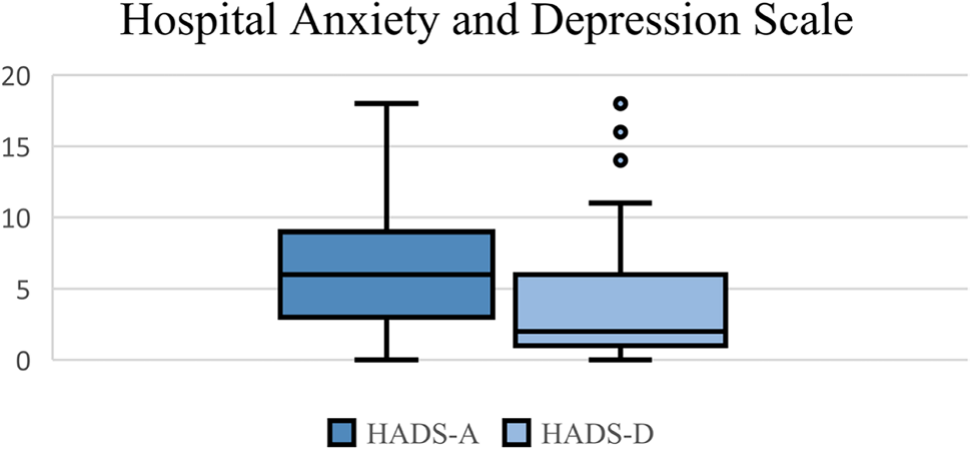
Box plot illustrating spread of scores in HADS A (anxiety) and D (depression) at 24 months. *N* = 117. Range of questionnaire: 0–21. Anxiety (HADS-A): median: 6 (IQR 3–9), range of data: 0–18, depression (HADS-D): median: 2 (IQR 1–6), range of data: 0–11

Out of the 237 patients in the study with breast symptom scores at 24 months, 120 (49.6%) returned the psychometric questionnaires on detailed pain assessment and pain perception. The association of psychometric questionnaire scores and breast symptoms at 24 months is shown in Table 3. Scores in the top quartile of the Short Form McGill Pain Questionnaire were associated with increased breast symptoms at 24 months (beta: 13.95, 95% CI: 6.98–20.92, p < 0.000) on multivariable analysis, although having scores in the top quartile of the Pain Sensitivity Questionnaire and Pain Catastrophising Scale were not. Higher scores in the West Haven-Yale Multidimensional Pain Inventory domains for life interference (3.80, 1.40–6.21, p = 0.002) and pain severity (4.97, 2.73–7.22, p < 0.001) were also associated with increased breast symptoms at 24 months on multivariable analysis, but the other domains assessing responses to pain and the effect on activities for daily living were not. Figure 3 shows the distribution of Hospital Anxiety and Depression Scale scores across the cohort. HADS-A scores indicating anxiety (≥ 8) were associated with increased breast symptoms (6.13, 0.17–12.09, p = 0.044) on univariable analysis; however, this association did not persist on multivariable analysis.

**Table 3:** Association of psychometric scores with breast symptom scores at 24 months. Values presented in this table are derived from simple linear regression with EORTC-QLQ-BR23 scores at 24 months. Multivariate regression was controlled for age, radiotherapy dose, axillary surgery type, BMI, smoking status, breast size, use of boost, and presence of hot spots. Sample size: 120 patients

	Univariable analysis			Multivariable analysis		
Variable	Beta co-efficient	Confidence interval	*P*-value	Beta co-efficient	Confidence interval	*P*-value
PSQ-Moderate (top quartile)	0.33	− 6.75–7.41	0.926	1.644	− 6.24–9.53	0.680
SF-MPQ A (top quartile)	**15.23**	**8.93**–**21.53**	**0.000**	**13.95**	**6.98–20.92**	**0.000**
PCS (top quartile)	5.27	− 1.57–12.11	0.130	5.00	− 2.57–12.58	0.193
HADS						
Anxiety (score ≥ 8)	**6.13**	**0.17–12.09**	**0.044**	4.94	− 1.83–11.71	0.151
Depression (score ≥ 8)	6.64	− 0.68–13.96	0.075	4.50	− 3.43–12.44	0.263
WHYMPI						
Interference	**4.14**	**1.97–6.31**	**0.000**	**3.80**	**1.40–6.21**	**0.002**
Support	0.89	− 1.30–3.08	0.423	1.53	− 0.97–4.03	0.228
Pain severity	**5.14**	**3.16–7.13**	**0.000**	**4.97**	**2.73–7.22**	** < 0.001**
Life control	** − 2.27**	** − 4.35** to − **0.18**	**0.033**	−1.12	− 3.49–1.25	0.349
Affective distress	**2.23**	**0.17–4.28**	**0.034**	1.78	− 0.52–4.07	0.127
Punishing responses	− 0.37	− 3.10–2.35	0.787	−0.35	− 3.20–2.50	0.807
Solicitous responses	0.87	− 0.97–2.71	0.353	1.77	− 0.30–3.84	0.092
Distracting responses	0.87	− 1.32–3.06	0.433	1.69	− 0.87–4.24	0.193
Household chores	− 0.21	− 2.58–2.16	0.860	0.12	− 2.41–2.66	0.924
Outdoor work	− 0.82	− 2.96–1.33	0.451	−0.94	− 3.30–1.43	0.433
Activities away from home	− 0.31	− 2.56–1.95	0.787	0.35	− 2.18–2.89	0.784
Social activities	0.83	− 1.30–2.95	0.443	1.57	− 0.85–3.99	0.201
General activities	− 0.28	− 3.23–2.66	0.849	0.31	− 2.94–3.57	0.849

*PSQ* Pain Sensitivity Questionnaire, *SF-MPQ* Short Form McGill Pain Questionnaire, *PCS* Pain Catastrophising Scale, *HADS* Hospital Anxiety and Depression Scale, *WHYMPI* West Haven-Yale Multidimensional Pain Inventory.

## Discussion

This study prospectively investigated patient-reported long-term breast pain and sensitivity up to two years following breast-conserving surgery and radiotherapy. It also explored the use of additional psychometric questionnaires to identify patients with increased long-term breast symptoms. The results of this research are pertinent to clinicians, as they strengthen the findings of previous research and suggest new potential risk factors to consider which have not been discussed previously.

In keeping with previous literature [8, 14, 28], median breast symptoms peaked on completion of radiotherapy and then decreased to at or below post-operative baseline by 24 months. However, this study showed that patients in the higher quartiles of scores at baseline continued to have elevated scores at 24 months, suggesting that it may be possible to identify distinct trajectories of patients with a higher level of baseline symptoms at risk of breast pain and sensitivity in the long-term, regardless of radiotherapy fractionation schedule. This could allow these patients to be specifically counselled about their increased risk of breast symptoms.

In terms of patient or treatment factors, younger women were more likely to develop more severe breast symptoms. Younger age has been shown to be a risk factor for breast pain and sensitivity in several studies of breast surgical patients [7], although the exact mechanism for this is not known. It is noted that pre-menopausal status was no longer significant in multivariate analysis when controlled for age, suggesting that any effect of pre-menopausal status is more likely to be related to younger age. Increasing BMI and breast size also appeared to be risk factors for adverse breast symptoms on univariate analysis, which correlates with previous reports, including the results of the Cambridge IMRT trial [15]. BMI is an important modifiable risk factor which could be addressed by patients prior to treatment. This is a particularly relevant given recent studies investigating the efficacy of GLP-1 drugs for breast cancer patients, including their use during treatment [29]. Smoking has been shown to be associated with increased risk of breast symptoms including skin complications in previous reports, which supports the results of this paper [15, 30]. It has been suggested that this increased risk may be due to desensitisation of nicotinic acetylcholine receptors, which is followed by hyperexcitability on withdrawal of nicotine [31]. Smoking is also known to cause oxidative stress, inflammation and impaired oxygen delivery, which can lead to poor tissue healing following damage from surgery or radiotherapy [32]. This finding is particularly important as smoking is a modifiable risk factor, so encouraging patients to cease smoking before commencing treatment may decrease rates of chronic breast symptoms. It is likely that the association between analgesic use before surgery and increased breast symptoms is related to pre-operative breast pain, which is an established risk factor for long-term breast pain [10]. Post-operative haematoseroma was also found to be associated with increased breast symptoms. There is little literature about the effect of post-operative complications in breast cancer patients, although there have been several cases of women who experienced a complete resolution of post-mastectomy pain syndrome symptoms following drainage of an axillary haematoma [33].

A large systematic review has found that IMRT is consistently associated with reduced rates of radiation dermatitis [34]. This finding was also demonstrated in the results of this study, with use of IMRT constituting a protective factor in comparison with using 3D conformal radiotherapy, independent of fractionation schedule. This highlights one of the advantages of IMRT over other more established radiotherapy techniques. Interestingly, in this study IMRT was only significant on multivariate analysis. It is possible that this may be due to the relation of IMRT with one of the co-factors used on multivariate analysis, such as age or axillary surgery. Unexpectedly, no significant difference was shown between fractionation schedules in terms of breast symptoms. Trials with larger proportions of patients receiving conventionally-fractionated radiotherapy have shown that hypofractionation leads to reduced breast symptoms [17, 35]. This may be explained by the fact that the majority (86.5%) of patients received the standard UK hypofractionated regimen of 40 Gy in 15 fractions, and therefore sample sizes were too limited to show the effect of differing schedules.

The levels of anxiety in our cohort were in keeping with previous investigations in breast cancer patients, which have found consistently higher anxiety sub-scores across all age groups [36]. HADS-A scores ≥ 8 (indicating a clinically significant case of anxiety) were associated with increased breast symptoms scores on univariate analysis. Due to the retrospective collection of psychometric scores, it is difficult to ascertain whether this anxiety predisposed to the increased breast scores, or whether those who have more severe symptoms are likely to be more anxious. However, these results do appear to support the hypothesis that anxiety may be associated with long-term breast symptoms, as suggested by previous authors [35]. A large meta-analysis found that cancer patients and their spouses were much more likely to be affected by anxiety than healthy controls, and this increased risk continued for ten or more years [37]. Depression rates were also increased in cancer patients and their spouses, although this only lasted for around two years. It is possible that depression levels had decreased in this cohort by the 24-month follow-up, which may explain why depression was not significantly associated with breast symptoms, as it has been in other studies.

One of the key strengths of this study was the use of an overall breast symptoms score from a validated PROs tool, rather than focussing on pain as one individual symptom as in previous studies [7, 30]. Incidence of breast symptoms is a complex and interlinked phenomenon and focussing on multiple symptoms provides a more holistic view of this. For example, breast oedema does not always present with the characteristic swelling seen in oedema of the extremities, and the only clinical signs may be skin changes, breast hardness and/or pain. This can lead to widely varying incidence rates, particularly when only one specific aspect of breast symptoms is asked about in questionnaires. This study used multilevel mixed modelling, which has been shown to be an effective technique for analysing quality-of-life data over time. It can cope well with missing data, and accounts for association of measures which were made on the same patient repeatedly, both of which are key considerations in studies that take place over multiple years [38]. This is supported by a paper published by the SISQOL consortium, which recommended use of linear mixed models for analysing QOL data in cancer randomised controlled trials [39].

To the authors' best knowledge, this is the first time that the West-Haven Yale Pain Inventory has been used to investigate pain in patients with breast cancer. This research also demonstrated that post-operative haematoseroma may increase women’s risk of developing chronic breast pain (causing an increase of 4.56 points on the EORTC-BR23, 95% CI 0.35–8.77, p = 0.034), which has only previously been discussed in a case report [40]. This study adds to the pool of data regarding patient and treatment factors related to breast pain and provides further opportunities for research. This may allow clinicians to identify women who are at higher risk of long-term side effects and used target supportive measures or counselling strategies. In a world where cancer rates are increasing and women are often being diagnosed earlier, the need to manage and pre-empt long-term side effects in cancer survivors is paramount.

### Limitations

This study included patients from one single centre, and ideally, this investigation should be replicated in other centres, which would provide a larger sample size for analysis. The patient cohort was also 92.4% White, meaning that sample sizes were small when comparing the incidence of breast symptoms in different ethnicities. Similarly, 88.2% of patients received IMRT, leaving a very small comparator group. Differences in planning approaches or patient selection may have contributed to the observed effect, limiting the generalisability of these findings. Future prospective studies would benefit from intentional design features aimed at achieving a more balanced representation of radiotherapy techniques. Such strategies include stratified or quota-based enrolment to ensure a predefined proportion of IMRT and non-IMRT patients; prospective treatment assignment frameworks or clinical trials (when ethically and practically feasible) that minimise passive imbalance; and multi-centre recruitment, particularly from centres that continue to use non-IMRT techniques.

A further limitation of this study is the timing of the “baseline” breast symptom score, which was collected prior to radiotherapy but after surgery. This means that the baseline PROs may have captured some postoperative symptoms such as pain, swelling, sensitivity, and functional limitation, if they persisted beyond eight to ten weeks. As postoperative complications (including hematoma or seroma) were themselves associated with worse long-term outcomes, it cannot be excluded that the baseline scores may reflect ongoing surgical or chemotherapy morbidity rather than pre-existing symptom burden or radiotherapy-related effects. This may complicate interpretation of both baseline differences and subsequent changes during follow-up, although our analysis clearly demonstrates distinct patient trajectories with symptoms that increase from pre-radiotherapy baseline.

The psychometric questionnaires were only administered at one time point (36 + months post-radiotherapy). If they had been used at each timepoint, including baseline, this would have allowed for an analysis of how psychometric scores change over time, as well as a prospective analysis of whether certain psychometric factors should be monitored prior to radiotherapy treatment. The use of questionnaire data also left this study vulnerable to selection bias, as patients who are suffering from increased symptoms are generally more likely to engage with studies and respond to questionnaires [41]. The response rate for the psychometric questionnaires was 49.6%, which is slightly lower than the mean response rate to postal questionnaires of approximately 60%, as reported by Asch et al. [42]. Use of telephone and written reminders have both been shown to increase questionnaire response rates, so for further studies these methods could be employed to improve return of questionnaires [43]. Steegers et al., who had a similar study design to this one, reported an initial response rate of 57% which increased to 68% after reminders were sent [44].

## Conclusions

Long-term breast symptoms after cancer treatment are an important area of research, particularly with increasing numbers of women living with and beyond cancer in the UK. This study demonstrates that the sub-set of patients with more severe breast symptoms at baseline continue to have more breast symptoms in the long-term. In addition, this study has identified several patient and treatment variables (smoking, use of IMRT, post-operative haematoseroma, younger age) that could be addressed in women at risk of developing these symptoms, such as through smoking cessation or providing more support to women with non-modifiable risk factors. The association with anxiety merits further investigation in a prospective study with questionnaires administered at baseline and at multiple timepoints after radiotherapy.

## Data Availability

The data from this study are available upon request (www.requite.eu) and may be utilized for various research purposes.
